# Morphological Characterization of Cherry Rootstock Candidates Selected from Central and East Black Sea Regions in Turkey

**DOI:** 10.1155/2013/916520

**Published:** 2013-12-18

**Authors:** Aysen Koc, Zumrut Celik, Mustafa Akbulut, Sukriye Bilgener, Sezai Ercisli, Mehmet Gunes, Resul Gercekcioglu, Ahmet Esitken

**Affiliations:** ^1^Department of Horticulture, Faculty of Agriculture and Natural Sciences, Bozok University, Yozgat, Turkey; ^2^Ministry of Food, Agriculture and Livestock, Ataturk Orman Ciftligi, Ankara, Turkey; ^3^Faculty of Agriculture and Natural Science, Recep Tayyip Erdogan University, Rize, Turkey; ^4^Department of Horticulture, Faculty of Agriculture, Ondokuz Mayis University, Samsun, Turkey; ^5^Department of Horticulture, Faculty of Agriculture, Ataturk University, Erzurum, Turkey; ^6^Department of Horticulture, Faculty of Agriculture, Gaziosmanpaşa University, Tokat, Turkey; ^7^Department of Horticulture, Faculty of Agriculture, Selcuk University, Konya, Turkey

## Abstract

The use of rootstocks particularly for sweet cherry cultivars is of great importance for successful and sustainable production. Choosing the right cherry rootstocks is just as important as choosing the right cultivar. In this study, 110 sweet cherry, 30 sour cherry, and 41 mahaleb types displaying rootstock potential for sweet cherry cultivars were selected from Central and East Black Sea Regions in Turkey. The morphologic characteristics of the studied genotypes were compared with the standard clonal rootstocks PHL-A, MaxMa 14, Montmorency, Weiroot 158, Gisela 5, Gisela 6, and SL 64. A total of 42 morphological UPOV characteristics were evaluated in the selected genotypes and clonal rootstocks. The obtained data were analyzed by using principal component analysis and it revealed that eigenvalues of the first 3 components were able to represent 36.43% of total variance. The most significant positive correlations of the plant vigor were determined with leaf blade length and petiole thickness. According to the diversity analysis of coefficients, the 05 C 002 and 08 C 039 genotypes were identified as being similar (6.66), while the 05 C 002 and 55 S 012 genotypes were determined as the most distant genotypes (325.84) in terms of morphology.

## 1. Introduction

Turkey produces 438.550 tons of sweet cherries and 182.234 tons of sour cherries annually playing an important role in world sweet and sour cherry production. According to recent statistics, Turkey takes the first place in both sweet cherry production and export [1]. In Turkey, the mahaleb (*Prunus mahaleb*), wild sweet cherry (*Prunus avium*), and wild sour cherry (*Prunus cerasus*) seedlings are widely used as rootstocks for both sweet and sour cherry cultivars. Ercisli et al. [2] reported that 40% of the sweet cherry production in Turkey is carried out with wild sweet cherry seedlings, 30% with mahaleb seedlings, and 30% with Gisela 5, Gisela 6, and SL 64 clone rootstocks. Sweet cherry scion cultivars have been selected over millennia for many reasons, while rootstocks have only recently received attention. Unlike scion cultivar development, evaluation of rootstock programs may require at least 10 years to be completed. However, improved new technologies have provided significant improvements in evaluation.

Genetic diversity (variation *within* species) is vital for the evolution of agricultural species and their adaptation to particular environments through a mixture of natural and human selection. In crop agriculture, for some species, this selection has led to the development of many thousands of “landraces” or “farmers” varieties. In addition to domesticated plants, wild species are important nutritionally and culturally to many people [3]. Germplasms collection and characterization are essential stages for breeding programs. Germplasms collection and characterization are generally performed by describing morphological characteristics. The international criteria of the International Union for the Protection of New Varieties of Plants (UPOV) and the International Plant Genetic Resources Institute (IPGRI) were created in order to remove unclear situation and to enable researchers to use common descriptive characteristics. The data from morphological traits were evaluated statistically by using principle component analysis (PCA), correlation, and morphological distance index.

Turkey is one of the most important genetic sources for cherries in the world and provides an important source of variation for plant breeding. However, as is the case with other species used in fruit production, our country does not have its own native cherry clonal rootstocks.

The aim of our study was to investigate genotypic variation among 110 sweet cherry, 30 sour cherry, and 41 mahaleb types selected among wild cherry populations in Central and East Black Sea Regions in Turkey that can potentially be used as rootstocks for cultivars in future.

## 2. Materials and Methods

**Figure 1:**
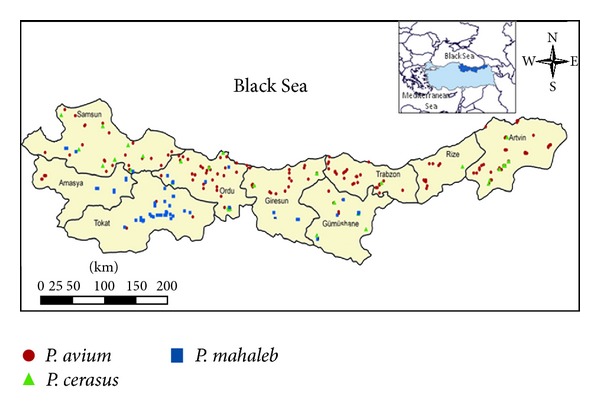
Map of the distribution for genotypes in Central and East Black Sea Regions in Turkey.

With an initial extensive survey studies, a total of 459 wild accessions were collected from Central and East Black Sea Regions in Turkey and were preserved at the Black Sea Agricultural Research Institute located in Samsun province during 2006–2009. The survey studies were conducted at Amasya, Artvin, Giresun, Gümüşhane, Ordu, Rize, Samsun, Tokat, and Trabzon provinces of Black Sea region. Selected wild genotypes (*P. avium*, *P. cerasus*, *P*. and *mahaleb*) were grafted by budding in the observation gardens. All types were identified using morphological characterization criteria of to UPOV (International Union for the Protection of New Varieties of Plants, Prunus Rootstocks 2002, TG/187/1-03.03.2007). Among 459 genotypes, we selected a total of 181 promising genotypes consist of 110 sweet cherry, 30 sour cherry, and 41 mahaleb and they were used for further analyses (Table 1). The morphologic characteristics of the studied genotypes were compared with PHL-A, MaxMa 14, Montmorency, Weiroot 158, Gisela 5, Gisela 6, and SL 64 worldwide reference clonal rootstocks. Morphological characteristics of the leaves were determined in July, while the morphological features of the shoots were determined in December. A total of 188 genotypes, including the selected genotypes and clonal rootstocks, were evaluated according to a total of 42 morphological and phenotypic characteristics (Table 2). Simple correlations, factor and cluster analyses, and scatter plots were prepared by using SPSS (version 20.0 for Windows). Factor analysis was performed by using the varimax factor rotating method. A dendrogram of the genetic similarities between the genotypes was compiled using the Ward method. The location data of selected genotypes was determined using GPS in the project area. These data were transferred in the GIS database and the distribution map was created using ArcGIS 9.2 software (Figure 1).

## 3. Results and Discussion

Great genetic diversity was observed among the wild sweet cherry, sour cherry, and mahaleb genotypes collected from Central and East Black Sea Region of Turkey. Several researchers have reported the morphological variation between some *Prunus subgenus cerasus* genotypes such as for sweet cherry (*P. avium*), sour cherry (*P. cerasus*), and mahaleb (*P. mahaleb*) [4–7].

**Figure 2:**
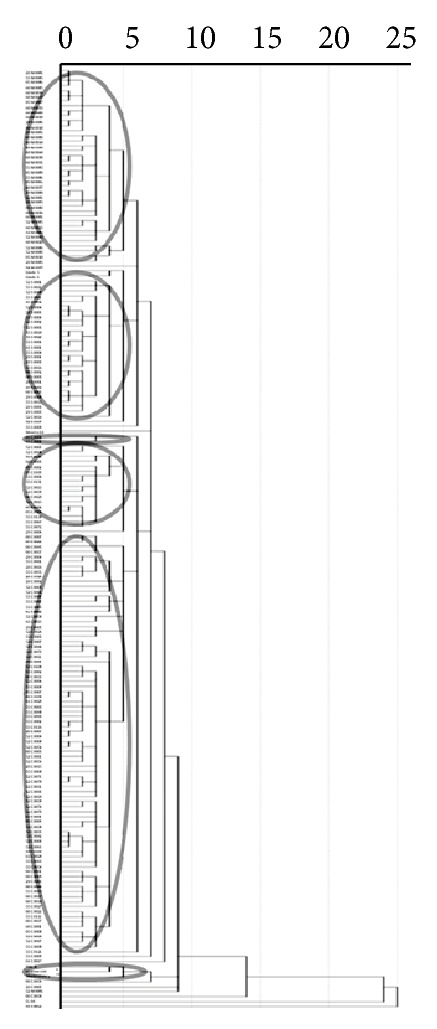
Cluster analysis for 188 genotypes/clonal rootstocks based on morphological data.

The morphological traits assessed showed a wide variation. Differences among cherries genotypes based on similarity of morphological characters are shown in Figure 2 using the hierarchical clustering. Unweighted pair group method with arithmetic mean cluster analysis revealed distance indexes between 6.66 and 325.84. A total of 188 genotypes including the selected genotypes and clonal rootstocks were examined for morphological distance. The closest sweet cherry rootstock candidates were 52 C 071 and 52 C 079 (12.79), while the most distant were 05 C 002 and 08 C 039 (184.29). The closest sour cherries were 53 S 001 and 61 S 001 (8.75), while the most distant were 08 S 002 and 55 S 021 (44.38). The closest mahalebs were 28 M 001 and 55 M 005 (6.66), while the most distant were 05 M 001 and 52 M 007 (72.14). The most distant genotypes among species were 05 C 002 and 55 S 012 (325.84). According to the analysis of the morphological index, all of the genotypes were distinguishable from one another. The dendrogram had eight main groups, which had twelve subgroups. The first group consisted of four subgroups and included one hundred seventy five genotypes and Gisela 5, Gisela 6, and Maxma 14 clonal rootstocks. The second group consisted of 08 C 056 genotype and PHL-A, Weiroot 158, and Montmorency clonal rootstocks. Other six groups consisted of only one genotype/clonal rootstock, respectively, 61 C 017, 28 C 005, 52 M 001, 08 C 039, SL 64, and 55 S 012.

Correlations between pomological traits were observed, but these data are not given in the tables in this paper. A characteristic such as plant vigor was positively correlated with leaf blade length (0.38), petiole length (0.34), and petiole thickness (0.36). One-year-old length of internodes was positively correlated with leaf blade length (0.60), petiole length (0.58), and petiole thickness (0.56), while it was negatively correlated with leaf shape (−0.40). One-year-old branching (at the end of summer) was negatively correlated with leaf blade length (−0.57), petiole length (−0.58), petiole thickness (−0.53), and one-year-old length of internodes (−0.51). Information regarding the associations and correlations between different plant characteristics are valuable for breeding programs.

Principal component analysis (PCA) was used to examine the variation of cherries genotypes/clonal rootstocks. Morphological characterization is necessary for the description and classification of germplasm and statistical methods like principal components analysis are useful tools for screening the accessions of a collection [8, 9]. It allows for visualization of the differences among the individuals, identification of possible groups, and relationships among individuals and variables [10]. The first thirteen axes accounted for 72.38% of the variability among 188 accessions (Table 3). The first PC axis accounted for 8.13% of the variation, whereas the second, third, and fourth axes accounted for 3.81%, 3.36%, and 2.75%, respectively. The first axis was mainly related to leaf blade length (0.91), petiole: thickness (0.84), petiole length (0.73), leaf blade: ratio length/width (0.71), and one-year-old shoot: length of internodes (0.70). The second axis was concerned with leaf: ratio length of leaf blade/length of petiole (−0.76). The third axis was mainly concerned with leaf: presence of stipules (0.87), petiole: presence of pubescence of upper side (0.87), and petiole: intensity of pubescence of upper side (0.79). The remaining ten axes were related to other leaf, shoot, and plant traits (Table 3).

The populations were grouped into seventeen clusters by cluster analysis. These are composed of six groups and eleven single genotypes. The different cherry genotypes identified based on the similarity of their morphological characteristics and their hierarchical clustering are shown in Figure 2. These seventeen groups can be considered as distinct germplasm pools (Figure 2). According to diversity analysis of coefficients, the 28 M 0001 and 55 M 0005 genotypes were found to be very similar (6.66), while the 05 C 0002 and 55 S 0012 genotypes were determined as the most distant genotypes (325.84) in terms of morphological variability. Shahi-Gharahlar et al. [11] reported that dendrogram obtained from morphological traits clearly distinguished the *Cerasus* subgenus genotypes from the other genotypes. Pérez-Sánchez et al. [4] suggested that a dendrogram obtained from morphological characteristics clearly showed the relationships between cultivars of sweet, sour, and duke cherries.

**Figure 3:**
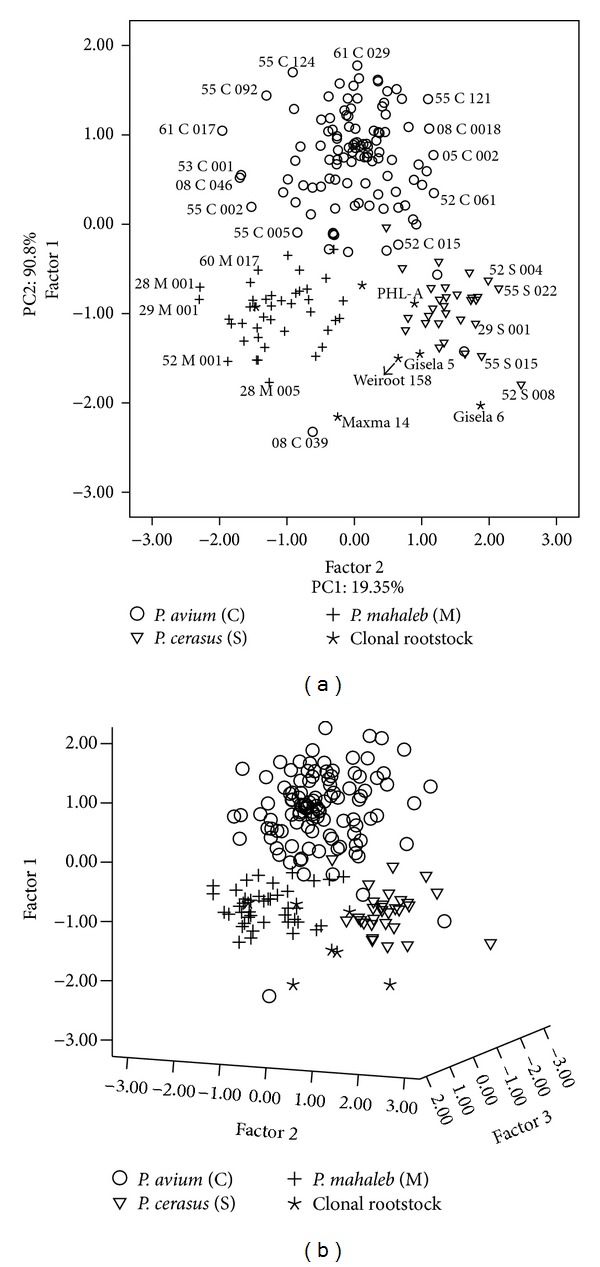
2D and 3D scatter diagrams of the relationships between the genotypes/clonal rootstocks (based on morphological traits).

The high total variance explained by the first three axes was shown in a 2D and 3D screen plot; each genotype/clonal rootstock was plotted based on its principal components score (the cumulative proportion of variance) for each of the first three axes (Figure 3).

## 4. Conclusions

As a result, it can be said that the studied genotypes are diverse and display great variations. The collection, evaluation, and characterization of Turkish cherries germplasm are a field of interest and are of economical and ecological importance. This provides rootstocks with good adaptations to diverse climatic and soil conditions of Turkey. The results may serve as a significant reference for the comparison of genetic resources, the characterization of cherry genotypes, and the cherry rootstock breeding programs to select the best parents with the highest variation. In conclusion, the genotypes evaluated in this study may be useful for both breeders and rootstock breeding programs.

## Figures and Tables

**Table 1:** The map data of types selected from Central and East Black Sea Regions in Turkey (C: sweet cherry, *P. avium*; S: sour cherry, *P. cerasus*; M: mahaleb, *P. mahaleb*; CA: *C. angustifolia*).

Accession	Collection area (province-district)	Altitude (m)
05 C 002	Amasya-Merkez	1002
05 C 003	Amasya-Taşova	640
05 C 004	Amasya-Taşova	640
05 C 005	Amasya-Gümüşhacıköy	821
05 C 006	Amasya-Gümüşhacıköy	826
05 C 007	Amasya-Gümüşhacıköy	875
05 C 009	Amasya-Gümüşhacıköy	956
05 M 001	Amasya-Merkez	449
05 M 006	Amasya-Merkez	846
05 M 007	Amasya-Taşova	1002
05 M 008	Amasya-Taşova	767
05 M 009	Amasya-Taşova	430
05 M 010	Amasya-Taşova	430

08 C 001	Artvin-Yusufeli	1554
08 C 005	Artvin-Yusufeli	1409
08 C 007	Artvin-Yusufeli	1445
08 C 008	Artvin-Yusufeli	1411
08 C 017	Artvin-Şavşat	1674
08 C 018	Artvin-Şavşat	1974
08 C 022	Artvin-Şavşat	1821
08 C 028	Artvin-Şavşat	1622
08 C 033	Artvin-Borçka	475
08 C 037	Artvin-Yusufeli	1488
08 C 039	Artvin-Yusufeli	1448
08 C 044	Artvin-Yusufeli	1499
08 C 045	Artvin-Yusufeli	1567
08 C 046	Artvin-Yusufeli	884
08 C 053	Artvin-Merkez	977
08 C 056	Artvin-Yusufeli	1084
08 C 057	Artvin-Yusufeli	1565
08 S 002	Artvin-Yusufeli	1560
08 S 003	Artvin-Yusufeli	675
08 S 005	Artvin-Yusufeli	1502

28 C 002	Giresun-Çanakçı	427
28 C 005	Giresun-Tirebolu	53
28 C 007	Giresun-Dereli	1014
28 C 015	Giresun-Yağlıdere	256
28 C 016	Giresun-Yağlıdere	68
28 C 020	Giresun-Bulancak	717
28 S 001	Giresun-Çanakçı	417
28 S 002	Giresun-Şebinkarahisar	1247
28 S 003	Giresun-Şebinkarahisar	1121
28 M 001	Giresun-Şebinkarahisar	1450
28 M 003	Giresun-Alucra	1464
28 M 005	Giresun-Şebinkarahisar	1270
28 CA 001	Giresun-Şebinkarahisar	1270
28 CA 002	Giresun-Şebinkarahisar	1217

29 C 001	Gümüşhane-Merkez	1199
29 C 003	Gümüşhane-Torul	1455
29 C 004	Gümüşhane-Kürtün	1276
29 C 005	Gümüşhane-Kürtün	1109
29 C 006	Gümüşhane-Torul	1065
29 S 001	Gümüşhane-Merkez	1287
29 S 003	Gümüşhane-Köse	1654
29 S 004	Gümüşhane-Şiran	1395
29 S 005	Gümüşhane-Torul	1043
29 M 001	Gümüşhane-Merkez	1281
29 M 004	Gümüşhane-Şiran	1229
29 M 006	Gümüşhane-Torul	1363
29 CA 001	Gümüşhane-Kelkit	1464
29 CA 002	Gümüşhane-Kelkit	1578
29 CA 003	Gümüşhane-Kelkit	1710
29 CA 004	Gümüşhane-Şiran	1329

52 C 004	Ordu-Aybastı	771
52 C 005	Ordu-Kabataş	623
52 C 007	Ordu-Mesudiye	1203
52 C 008	Ordu-Gölköy	919
52 C 009	Ordu-Kabadüz	189
52 C 011	Ordu-Fatsa	610
52 C 013	Ordu-Korgan	1525
52 C 014	Ordu-Korgan	1162
52 C 015	Ordu-Kumru	579
52 C 019	Ordu-Kumru	730
52 C 026	Ordu-Kabataş	613
52 C 029	Ordu-Çamaş	155
52 C 030	Ordu-Çamaş	474
52 C 031	Ordu-Çatalpınar	359
52 C 035	Ordu-Çatalpınar	640
52 C 038	Ordu-Mesudiye	1289
52 C 039	Ordu-Mesudiye	1072
52 C 042	Ordu-Gölköy	829
52 C 046	Ordu-Gürgentepe	1015
52 C 050	Ordu-Gürgentepe	1021
52 C 054	Ordu-Ulubey	513
52 C 056	Ordu-Merkez	219
52 C 061	Ordu-Kabadüz	406
52 C 063	Ordu-Gülyalı	195
52 C 065	Ordu-Gülyalı	357
52 C 071	Ordu-Merkez	524
52 C 073	Ordu-Perşembe	229
52 C 074	Ordu-Perşembe	110
52 C 078	Ordu-Perşembe	297
52 C 079	Ordu-Ünye	374
52 C 081	Ordu-Çaybaşı	536
52 C 087	Ordu-İkizce	341
52 C 090	Ordu-Akkuş	1097
52 C 091	Ordu-Akkuş	1198
52 C 093	Ordu-Akkuş	965
52 C 096	Ordu-Fatsa	331
52 C 100	Ordu-Ünye	354
52 S 001	Ordu-Kabataş	450
52 S 002	Ordu-Çamaş	329
52 S 003	Ordu-Mesudiye	1138
52 S 004	Ordu-Mesudiye	1302
52 S 005	Ordu-Mesudiye	1133
52 S 006	Ordu-Perşembe	0
52 S 007	Ordu-Çaybaşı	430
52 S 008	Ordu-Akkuş	1102
52 M 001	Ordu-Kabataş	503
52 M 003	Ordu-Mesudiye	1350
52 M 005	Ordu-Merkez	376
52 M 006	Ordu-Akkuş	1258
52 M 007	Ordu-Akkuş	1125
52 M 008	Ordu-Akkuş	1223
52 M 009	Ordu-Akkuş	1064

53 C 001	Rize-İkizdere	780
53 C 002	Rize-İkizdere	968
53 C 005	Rize-İkizdere	838
53 C 006	Rize-İkizdere	701
53 C 008	Rize-Güneysu	518
53 C 009	Rize-Çayeli	798
53 S 001	Rize-Çamlıhemşin	1315

55 C 002	Samsun-Ladik	958
55 C 005	Samsun-Ladik	754
55 C 015	Samsun-Vezirköprü	749
55 C 027	Samsun-Merkez	722
55 C 040	Samsun-Terme	427
55 C 049	Samsun-Asarcık	1013
55 C 054	Samsun-Asarcık	810
55 C 055	Samsun-Havza	1033
55 C 060	Samsun-Çarşamba	658
55 C 065	Samsun-Ondokuzmayıs	247
55 C 067	Samsun-Bafra	623
55 C 072	Samsun-Alaçam	683
55 C 080	Samsun-Yakakent	151
55 C 081	Samsun-Yakakent	246
55 C 083	Samsun-Terme	287
55 C 092	Samsun-Kavak	753
55 C 093	Samsun-Kavak	765
55 C 105	Samsun-Vezirköprü	659
55 C 111	Samsun-Bafra	431
55 C 116	Samsun-Ayvacık	691
55 C 121	Samsun-Salıpazarı	1097
55 C 124	Samsun-Tekkeköy	126
55 C 131	Samsun-Çarşamba	328
55 C 134	Samsun-Bafra	510
55 S 004	Samsun-Havza	821
55 S 008	Samsun-Ondokuzmayıs	236
55 S 011	Samsun-Yakakent	685
55 S 012	Samsun-Ayvacık	173
55 S 015	Samsun-Çarşamba	168
55 S 016	Samsun-Ladik	909
55 S 019	Samsun-Ayvacık	630
55 S 021	Samsun-Merkez	560
55 S 022	Samsun-Asarcık	779
55 M 001	Samsun-Vezirköprü	351
55 M 003	Samsun-Havza	352
55 M 005	Samsun-Vezirköprü	351
55 M 006	Samsun-Vezirköprü	332
55 M 009	Samsun-Ayvacık	700

60 C 001	Tokat-Reşadiye	1152
60 C 005	Tokat-Pazar	1180
60 M 001	Tokat-Merkez	655
60 M 002	Tokat-Merkez	673
60 M 005	Tokat-Almus	1074
60 M 008	Tokat-Almus	1004
60 M 010	Tokat-Almus	1004
60 M 014	Tokat-Almus	805
60 M 015	Tokat-Almus	796
60 M 016	Tokat-Almus	802
60 M 017	Tokat-Almus	789
60 M 019	Tokat-Almus	817
60 M 028	Tokat-Niksar	710
60 M 030	Tokat-Niksar	672
60 M 031	Tokat-Merkez	676
60 M 033	Tokat-Merkez	834
60 M 036	Tokat-Pazar	1013
60 M 037	Tokat-Pazar	1044
60 M 044	Tokat-Merkez	783

61 C 002	Trabzon-Araklı	215
61 C 015	Trabzon-Maçka	992
61 C 017	Trabzon-Akçaabat	1714
61 C 020	Trabzon-Düzköy	585
61 C 022	Trabzon-Tonya	733
61 C 029	Trabzon-Beşikdüzü	63
61 S 001	Trabzon-Araklı	468
61 S 002	Trabzon-Merkez	10

**Table 2:** Morphological characteristics used for the characterization of cherry types.

Plant		
Vigor	PV	Weak (3), medium (5), strong (7)
Habit	PH	Upright (1), spreading (3), drooping (5)
Branching	PB	Weak (3), medium (5), strong (7)
One-year-old shoot		
Thickness	ST	Thin (3), medium (5), thick (7)
Length	SL	(cm)
Length of internodes	SIL	Short (3), medium (5), long (7)
First branch height	FBH	(cm)
Branch angle	BA	(°)
Pubescence (upper third)	SP	Absent (1), present (9)
Number of lenticels	SLN	Few (3), medium (5), many (7)
Anth. coloration of apex	SA	Absent or very weak (1), weak (3), medium (5), strong (7), very strong (9)
Position of vegetative bud	SBP	Adpressed (1), slightly held out (2), markedly held out (3)
Size of vegetative bud	SBS	Small (3), medium (5), large (7)
Shape of apex of vegetative bud	BAS	Acute (1), obtuse (2), rounded (3)
Size of vegetative bud support	BSS	Small (3), medium (5), large (7)
Branching (at the end of summer)	SB	Number of branching
Intensity of anthocyanin coloration of young leaf	LAI	Weak (3), medium (5), strong (7)
Leaf		
Length	LL	(cm)
Width	LW	(cm)
Ratio length/width	RLW	Very small (1), small (3), medium (5), large (7), very large (9)
Shape	LS	Narrow elliptic (1), elliptic (2), circular (3), ovate (4), obovate (5)
Angle of apex (excluding tip)	LAA	Acute (1), right-angled (2), obtuse (3)
Length of tip	LTL	Short (3), medium (5), long (7)
Shape of base	LBS	Acute (1), obtuse (2), truncate (3)
Color of upper side	LUC	light green (1), dark green (2), red (3), reddish brown (4)
Glossiness of upper side	LUG	Weak (3), medium (5), strong (7)
Pubescence of lower side at apex	LLP	Weak (3), medium (5), strong (7)
Incisions of margin	LMI	Only crenate (1), both crenate and serrate (2), only serrate (3)
Depth of incisions of margin	LMID	Shallow (3), medium (5), deep (7)
Petiole length	PL	Short (3), medium (5), long (7)
Petiole presence of pubescence	PUP	Absent (1), present (9)
Petiole intensity of pubescence	PUPI	Weak (3), medium (5), strong (7)
Petiole depth of groove	PGD	Shallow (3), medium (5), deep (7)
Petiole thickness	PT	(cm)
Ratio length of leaf/petiole	RLPL	small (3), medium (5), large (7)
Presence of stipules	LSP	Absent (1), present (9)
Stipule length	STL	Short (3), medium (5), long (7)
Presence of nectaries	LN	Absent (1), present (9)
Predominant number of nectaries	LNN	One (1), two (2), more than two (3)
Position of nectaries	LNP	predominantly on base of blade (1), equally distributed on base of blade and petiole (2), predominantly on petiole (3)
Nectary color	NC	Gren (1), yellow (2), red (3), violet (4)
Nectary shape	NS	Round (1), reniform (2)

**Table 3:** Eigenvalues and proportions of variance described by the 13 principal components that correspond to eigenvalues greater than 1.

	PC axis												
	1	2	3	4	5	6	7	8	9	10	11	12	13
Eigenvalues	8,13	3,81	3,36	2,75	1,93	1,66	1,55	1,42	1,34	1,25	1,14	1,06	1,01
Explained proportion of variation (%)	19,35	9,08	8,00	6,54	4,60	3,96	3,68	3,37	3,19	2,98	2,71	2,52	2,41
Cumulative proportion of variation (%)	19,35	28,43	36,43	42,97	47,56	51,52	55,20	58,57	61,76	64,74	67,46	69,98	72,38

Characters	Eigen vectors												

Leaf blade: shape	**−0,69**	0,33	0,10	0,10	0,17	−0,01	0,10	0,00	0,04	0,13	0,09	−0,10	−0,11
Leaf blade: angle of apex	−0,36	**0,55**	0,14	−0,22	−0,06	−0,02	−0,07	0,07	−0,10	−0,14	0,09	0,32	0,15
Leaf blade: length of tip	0,47	**−0,50**	−0,05	0,09	−0,08	0,05	0,04	0,14	−0,13	0,03	0,05	−0,32	−0,11
Leaf blade: shape of base	−0,28	**0,55**	0,13	−0,15	0,26	−0,28	0,03	−0,21	0,09	0,05	0,14	0,00	0,15
Leaf blade: color of upper side	0,24	−0,16	−0,34	0,13	0,15	−0,30	−0,23	**0,39**	0,19	−0,19	−0,02	−0,21	0,24
Leaf blade: glossiness of upper side	−0,23	−0,12	0,22	−0,01	0,12	0,22	**0,47**	−0,41	−0,12	0,04	−0,08	−0,01	0,20
Leaf blade: pubescence of lower side at apex	0,36	0,19	−0,21	−0,04	0,03	−0,06	0,16	0,24	0,05	**0,37**	0,34	0,04	0,33
Young shoot intensity of anth. coloration of young leaf	**0,49**	−0,01	0,44	0,22	−0,06	0,04	−0,22	−0,26	−0,18	−0,11	0,35	−0,05	−0,09
Leaf blade: incisions of margin	**0,48**	−0,10	−0,16	0,09	0,10	0,15	0,13	−0,26	0,28	0,40	−0,12	−0,24	−0,10
Leaf blade: depth of incisions of margin	0,42	−0,12	−0,13	−0,10	**0,51**	−0,25	−0,10	−0,04	0,19	−0,01	−0,09	−0,01	0,23
Leaf: presence of stipules	−0,05	−0,21	**0,87**	−0,21	−0,06	−0,03	−0,02	0,21	0,05	0,12	−0,13	0,05	0,10
Petiole: presence of pubescence of upper side	−0,03	−0,19	**0,87**	−0,25	−0,05	0,00	−0,01	0,19	0,06	0,15	−0,14	0,07	0,10
Petiole: intensity of pubescence of upper side	0,16	−0,34	**0,79**	−0,26	0,10	0,00	−0,04	0,10	0,12	0,13	−0,14	0,03	0,06
Petiole: depth of Groove	**0,54**	−0,23	−0,06	−0,08	0,41	−0,09	−0,04	0,04	0,03	−0,18	−0,08	0,21	0,14
Leaf: presence of nectaries	0,09	0,18	0,27	−0,19	0,02	0,04	**0,56**	0,22	0,04	−0,32	0,18	−0,37	−0,09
Leaf: predominant number of nectaries	**0,53**	0,30	0,13	−0,02	0,10	−0,15	0,33	0,06	−0,08	−0,19	−0,05	−0,31	0,17
Leaf: position of nectaries	0,26	**0,43**	−0,06	−0,20	−0,31	0,09	0,01	0,37	−0,37	−0,01	0,24	0,07	0,16
Nectary: color	**0,67**	−0,12	0,03	−0,12	0,05	−0,06	−0,04	0,13	−0,08	−0,07	0,02	−0,26	−0,10
Nectary: shape	0,15	0,20	0,08	−0,08	0,06	−0,17	0,30	0,10	0,42	**−0,44**	0,09	0,24	−0,25
Leaf blade: length	**0,91**	0,10	−0,03	0,08	0,06	−0,03	−0,02	−0,09	−0,04	−0,08	−0,14	0,14	−0,02
Leaf blade: width	**0,68**	0,45	0,01	0,15	0,15	−0,08	0,04	−0,03	0,03	−0,04	−0,16	0,16	−0,11
Leaf blade: ratio length/width	**0,71**	−0,42	−0,09	−0,07	−0,10	0,09	−0,08	−0,09	−0,11	−0,10	−0,04	0,05	0,12
Petiole: length	**0,73**	0,54	0,08	0,00	0,04	−0,07	−0,01	−0,14	0,00	0,10	−0,09	0,02	−0,07
Leaf: ratio length of leaf blade/length of petiole	0,07	**−0,76**	−0,12	0,12	0,01	0,10	−0,03	0,11	−0,05	−0,24	0,00	0,11	0,08
Petiole: thickness	**0,84**	0,13	−0,01	0,08	0,08	0,02	−0,01	−0,06	−0,02	0,05	−0,11	0,10	−0,03
Stipule: length	**0,50**	−0,19	0,29	−0,08	0,03	−0,04	−0,07	0,00	0,33	0,01	0,40	0,01	−0,20
Plant: vigor	**0,43**	0,12	−0,04	0,02	0,10	0,51	0,18	−0,11	0,06	−0,10	0,16	0,08	0,21
Plant: habit	−0,15	0,15	−0,09	−0,04	0,27	**0,59**	−0,01	0,15	0,32	0,07	−0,03	−0,04	−0,10
Plant: branching	−0,16	0,07	−0,05	−0,02	0,37	**0,64**	−0,05	0,34	0,02	−0,01	0,02	0,05	−0,05
Pubescence of shoot	−0,11	−0,08	0,05	−0,10	**0,61**	−0,03	0,14	−0,10	−0,40	−0,02	0,01	0,04	0,05
Anthocyanin coloration of apex	**0,53**	−0,07	0,38	0,19	−0,07	0,18	−0,25	−0,23	−0,13	−0,17	0,33	0,01	−0,05
One-year-old shoot: pos. of veg. bud in relation to shoot	0,21	**−0,43**	−0,14	0,14	0,09	−0,12	0,32	−0,05	−0,08	0,33	0,35	0,10	0,12
One-year-old shoot: size of vegetative bud	0,08	0,25	0,04	0,28	**−0,50**	0,08	−0,06	−0,03	0,34	−0,01	−0,09	−0,18	0,42
One-year-old shoot: shape of apex of vegetative bud	0,01	**−0,28**	0,05	0,25	−0,24	−0,26	0,52	−0,01	0,03	0,07	−0,06	0,17	−0,10
One-year-old shoot: size of vegetative bud support	0,17	−0,32	−0,16	0,31	−0,10	−0,04	0,20	0,25	0,19	0,15	0,14	**0,38**	−0,08
One-year-old shoot: length	−0,09	0,08	0,43	**0,68**	0,21	0,04	−0,04	−0,04	0,06	−0,07	0,08	−0,01	0,22
One-year-old shoot: thickness	−0,10	0,27	0,24	**0,75**	0,05	−0,04	−0,18	−0,01	0,21	0,04	0,08	−0,05	−0,02
One-year-old shoot: length of internode	**0,70**	0,27	−0,09	0,07	−0,09	0,19	0,05	0,20	−0,07	0,18	−0,14	0,10	0,03
One-year-old shoot: number of lenticels	0,12	0,11	−0,04	**−0,55**	0,21	−0,20	−0,25	0,03	0,08	0,31	0,27	−0,09	−0,15
One-year-old shoot: branching	**−0,66**	−0,24	0,04	0,39	0,32	−0,09	−0,06	0,10	−0,03	−0,06	0,10	−0,04	0,05
First branch height	**0,56**	0,32	0,18	0,27	0,01	−0,02	0,02	0,22	−0,21	0,16	−0,19	0,00	−0,18
Branch angle	−0,20	0,14	0,17	**0,61**	0,20	−0,14	0,04	0,30	−0,31	0,13	−0,07	−0,04	−0,16

## References

[1] Food and Agricultural Organization (FAO) http://faostat.fao.org/site/567/DesktopDefault.aspx?PageID=567.

[2] Ercisli S, Esitken A, Orhan E, Ozdemir O (2006). Rootstocks used for temperate fruit trees in Turkey: an overview. *Sodininkyste ir Darzininkyste*.

[3] Cromwell E Agricultural, biodiversity and livelihood: issue and entry point: final report. http://www.odi.org.uk/sites/odi.org.uk/files/odi-assets/publications-opinion-files/8286.pdf.

[4] Pérez-Sánchez R, Gómez-Sánchez MA, Morales-Corts R (2008). Agromorphological characterization of traditional Spanish sweet cherry (*Prunus avium* L.), sour cherry (*Prunus cerasus* L.) and duke cherry (*Prunus × gondouinii* Rehd.) cultivars. *Spanish Journal of Agricultural Research*.

[5] Khadivi-Khub A, Zamani Z, Bouzari N (2008). Evaluation of genetic diversity in some Iranian and foreign sweet cherry cultivars by using RAPD molecular markers and morphological traits. *Horticulture Environment and Biotechnology*.

[6] Moghadam EG, Khalighi A (2007). Relationship between vigor of Iranian *Prunus mahaleb* L. selected dwarf rootstocks and some morphological characters. *Scientia Horticulturae*.

[7] Rakonjac V, Akšić MF, Nikolić D, Milatović D, Čolić S (2010). Morphological characterization of “Oblačinska” sour cherry by multivariate analysis. *Scientia Horticulturae*.

[8] Cantini C, Cimato A, Sani G (1999). Morphological evaluation of olive germplasm present in Tuscany region. *Euphytica*.

[9] Badenes ML, Martínez-Calvo J, Llácer G (2000). Analysis of a germplasm collection of loquat (*Eriobotrya japonica* Lindl.). *Euphytica*.

[10] Martínez-Calvo J, Gisbert AD, Alamar MC (2008). Study of a germplasm collection of loquat (*Eriobotrya japonica* Lindl.) by multivariate analysis. *Genetic Resources and Crop Evolution*.

[11] Shahi-Gharahlar A, Zamani Z, Fatahi MR, Bouzari N (2010). Assessment of morphological variation between some Iranian wild *Cerasus* sub-genus genotypes. *Horticulture Environment and Biotechnology*.

